# Characteristics of hallucinatory-paranoid disorders in patients with vascular dementia of different stages of development

**DOI:** 10.1192/j.eurpsy.2021.632

**Published:** 2021-08-13

**Authors:** N. Maruta, K. Shevchenko-Bitenskiy

**Affiliations:** 1 Borderline Psychiatry, “Institute of Neurology, Psychiatry and Narcology of NAMS of Ukraine” SI, Kharkiv, Ukraine; 2 Psychiatry, Narcology And Psychology, Odesa National Medical University, Odesa, Ukraine

**Keywords:** vascular dementia, hallucinatory-paranoid disorders

## Abstract

**Introduction:**

The most frequent and severe non-cognitive disorders in dementia are hallucinatory-paranoid disorders (HPD), which cause social dysfunction and financial burden of this pathology.

**Objectives:**

To study the features of HPD in vascular dementia (VD), an approach using clinical-psychopathological, psychometric, psychodiagnostic and mathematical-statistical methods was used.

**Methods:**

The study was based on the examination of 75 patients with HPD in VD and 63 patients with VD without HPD.

**Results:**

In patients with VD in the middle stage of development in the structure of clinical manifestations was dominated by frequent paranoid and paranoid disorders (in 75.6% of patients, p <0.05) with a systemic delusional plot (in 70.1% of patients, p <0.01) material damage, robbery, theft (in 26.8% of patients, p <0.01), relationships (in 21.9% of patients, p <0.01) and jealousy (in 17.1% of patients, p <0, 01), which ran in the form of paranoid delusional disorder (63.4%), acute paranoia (12.2%) and hallucinations (24.4%). In patients with VD in the late stage of development, the clinical and psychopathological structure of GPR was characterized by a predominance of frequent, hallucinatory disorders (82.4% of patients, p <0.01) in the form of healthy (23.5%, p <0.1), tactile (20.6%, p <0.01) and auditory (26.5%, p <0.5) hallucinations, which took the form of hallucinations (44.2%, p <0.05), confusion (61.5 %, p <0.05) and paranoid delusional disorder (17.6%, p <0.01).

**Conclusions:**

The study of the clinical and psychopathological structure of HPD in patients with dementia of different stages of development revealed their dependence on the stage of development of the pathological process.

